# Optimizing Early-stage Clinical Pharmacology Evaluation to Accelerate Clinical Development of Giredestrant in Advanced Breast Cancer

**DOI:** 10.1158/2767-9764.CRC-23-0324

**Published:** 2023-12-15

**Authors:** Vikram Malhi, Priya Agarwal, Mary R. Gates, Lichuan Liu, Jianshuang Wang, Tom De Bruyn, Scott Lam, Jennifer Eng-Wong, Pablo Perez-Moreno, Ya-Chi Chen, Jiajie Yu

**Affiliations:** 1 Clinical Pharmacology, Genentech, Inc., South San Francisco, California.; 2 Early Clinical Development, Genentech, Inc., South San Francisco, California.; 3 Drug Metabolism and Pharmacokinetics, Genentech, Inc., South San Francisco, California.; 4 BioAnalytical Sciences, Genentech, Inc., South San Francisco, California.; 5 Product Development Oncology, Genentech, Inc., South San Francisco, California.

## Abstract

**Purpose:**

We describe the clinical pharmacology characterization of giredestrant in a first-in-human study.

**Experimental Design:**

This phase Ia/Ib dose-escalation/-expansion study (NCT03332797) evaluated the safety, pharmacokinetics, pharmacodynamics, and preliminary antitumor activity of giredestrant in estrogen receptor–positive HER2-negative locally advanced/metastatic breast cancer. The single-agent dose-escalation stage evaluated giredestrant 10, 30, 90, or 250 mg once daily. The dose-expansion stage evaluated single-agent giredestrant at 30, 100, and 250 mg once daily. Dose-escalation and -expansion phases also evaluated giredestrant 100 mg combined with palbociclib 125 mg.

**Results:**

Following single-dose oral administration, giredestrant was rapidly absorbed and generally showed a dose-proportional increase in exposure at doses ranging from 10 to 250 mg. At the 30 mg clinical dose, maximum plasma concentration was 266 ng/mL (50.1%) and area under the concentration–time curve from 0 to 24 hours at steady state was 4,320 ng·hour/mL (59.4%). Minimal giredestrant concentrations were detected in urine, indicating that renal excretion is unlikely to be a major elimination route for giredestrant. Mean concentration of 4beta-hydroxycholesterol showed no apparent increase over time at both the clinical dose (30 mg) and a supratherapeutic dose (90 mg), suggesting that giredestrant may have low CYP3A induction potential in humans. No clinically relevant drug–drug interaction was observed between giredestrant and palbociclib. Giredestrant exposure was not affected by food and was generally consistent between White and Asian patients.

**Conclusions:**

This study illustrates how the integration of clinical pharmacology considerations into early-phase clinical trials can inform the design of pivotal studies and accelerate oncology drug development.

**Significance:**

This work illustrates how comprehensive clinical pharmacology characterization can be integrated into first-in-human studies in oncology. It also demonstrates the value of understanding clinical pharmacology attributes to inform eligibility, concomitant medications, and combination dosing and to directly influence late-stage trial design and accelerate development.

## Introduction

The clinical development journey of novel pharmaceuticals is a complex multistage process, which continues to evolve as our understanding of disease biology deepens, new therapeutic entities and classes of drugs are designed, and regulatory mechanisms adapt to the changing therapeutic landscape. Traditionally, oncology clinical development began with dose-escalation studies that aimed to identify the maximum tolerated dose in small cohorts of patients (1–3) and provided a preliminary assessment of activity and tolerability. However, increasing the dose for nonchemotherapy agents does not always increase activity. An inappropriately high dose could saturate efficacy and bring unnecessary toxicity. To provide an optimized risk:benefit profile for patients, clinical pharmacology characterization can be a useful tool to elucidate the exposure characteristics of an investigational drug and understand how exposure affects clinical outcomes.

One of the objectives of clinical pharmacology is to characterize pharmacokinetics by understanding the absorption, distribution, metabolism, and excretion pathways of the drug in the body. Another major objective is to determine how drug concentrations are altered by intrinsic and extrinsic factors. Intrinsic factors are inherent to a person and include age, sex, race, genomics, and organ function, whereas extrinsic factors are external influences that may affect drug exposure, such as concomitant medications or the type of food ingested around the time of drug administration. Finally, the main goal of clinical pharmacology is to justify the dose based on the relationship between exposure and biological response, which can be assessed in terms of efficacy, safety, or pharmacodynamic biomarkers.

Clinical pharmacology findings generated in early studies can inform late-stage study designs. For example, insight into potential interactions with food and concomitant medications can guide decisions on drug administration schedules and prohibited or permitted concomitant medications (4). Likewise, an understanding of risk related to intrinsic factors defined by age, race, organ function, etc., can guide eligibility criteria for phase III trials (4). For instance, patients with renal impairment may be more susceptible to toxicities because of altered pharmacokinetics, and potentially require dose modification of drugs eliminated predominantly by the kidneys. Clinically relevant differences between racial or ethnic groups concerning drug metabolism and drug exposure may require dose adjustments (5). Incorporating these clinical pharmacology objectives into early clinical studies designed to provide preliminary evidence of efficacy and safety in the target population can potentially accelerate late-stage development.

In this article, we describe the clinical pharmacology characterization of giredestrant, a highly potent nonsteroidal oral selective estrogen receptor (ER) antagonist and degrader (6) in the context of early-stage clinical development. Giredestrant was designed to optimize ER antagonism and degradation, while minimizing off-target toxicity (7). Several oral selective estrogen receptor degraders (SERDs) are in clinical development, all with distinct physicochemical and pharmacokinetics properties. Variations in these characteristics, such as unfavorable pharmacokinetic attributes [including nonlinear pharmacokinetics, food effects, or drug–drug interactions (DDIs) with combination partners] may affect dosing, efficacy, and safety (7).

This first-in-human phase Ia/Ib dose-escalation/-expansion study was designed to evaluate the safety, pharmacokinetics, pharmacodynamics, and preliminary antitumor activity of giredestrant. In particular, the pharmacokinetics were evaluated by characterizing the absorption, distribution, metabolism, and excretion of giredestrant, and generated signal-seeking data on the impact of certain intrinsic factors (including race and organ dysfunction) and extrinsic factors (including the effect of food and DDIs) on giredestrant exposure. The primary results from this study have been reported elsewhere (8). Here we focus on aspects of the study that enabled us to conduct an integrated clinical pharmacology assessment to inform better our late-stage development decisions.

## Materials and Methods

### Study Design

This multicenter nonrandomized open-label dose-escalation and -expansion phase Ia/Ib study (clinicaltrials.gov NCT03332797; GO39932) evaluated the safety, pharmacokinetics, pharmacodynamics, and preliminary antitumor activity of giredestrant alone and in combination with palbociclib in patients with ER-positive (ER^+^) HER2-negative locally advanced/metastatic breast cancer. The study design (Supplementary Fig. S1) has been described in detail by Jhaveri and colleagues (8), together with the clinical results. In brief, eligible patients had advanced or metastatic ER^+^/HER2-negative breast cancer that had recurred or progressed while being treated with adjuvant endocrine therapy (ET) for ≥24 months and/or ET in the incurable, locally advanced, or metastatic setting and derived a clinical benefit from therapy (i.e., tumor response or stable disease for ≥6 months); had not received any other ET, targeted therapy, or chemotherapy within the preceding 2 weeks; and were postmenopausal women (or premenopausal/perimenopausal women simultaneously receiving luteinizing hormone-releasing hormone agonists). To enroll a racially diverse population, the study was performed globally at sites in Europe, North America, Asia, and Australia (Supplementary Table S1).

In the single-agent dose-escalation stage, giredestrant was administered orally once daily on days 1–28 of each 28-day cycle at 10, 30, 90, or 250 mg. A single dose was administered on cycle 1 day −7, followed by a 7-day pharmacokinetic lead-in for all single-agent giredestrant dose-escalation cohorts. In addition, a combination cohort in the dose-escalation stage explored giredestrant 100 mg once daily (days 1–28) in combination with palbociclib 125 mg once daily (days 1–21), repeated every 28 days. In the expansion stage, single-agent expansion cohorts evaluated giredestrant at doses of 30, 100, and 250 mg once daily. A combination expansion cohort evaluated giredestrant 100 mg with palbociclib 125 mg.

### Pharmacokinetic Assessments

In the single-agent dose-escalation stage, pharmacokinetic samples were collected at cycle 1 day −7 predose and then at 0.5, 1, 1.5, 2, 3, 4, 6, 8, 24, 28, 72, 96, and 168 hours after dosing to characterize the single-dose pharmacokinetic profile and estimate the terminal half-life. Samples to determine the steady-state pharmacokinetic profile were collected at cycle 2 day 1 (after 28 days of daily giredestrant) predose and at 0.5, 1, 1.5, 2, 3, 4, 6, and 8 hours after dosing. Metabolite identification was conducted following the first dose (cycle 1 day −7) and at steady state (cycle 2 day 1) in 6 patients (3 patients at 90 mg and 3 at 250 mg). Plasma samples were pooled across patients and timepoints.

The cytochrome P450 (CYP) 3A induction potential of giredestrant was evaluated *in vivo* by assessing 4beta-hydroxycholesterol (4β-HC), an endogenous biomarker formed by CYP3A metabolism in humans (9). 4β-HC concentration was measured at cycle 1 day −7, cycle 2 day 1, cycle 3 day 1, and cycle 4 day 1 predose in 7 patients (5 patients at 30 mg and 2 at 90 mg). Analysis was conducted *ad hoc*, in compliance with the informed consent, in patients from whom there was sufficient plasma volume remaining for the assessment after completion of prespecified analyses.

Giredestrant concentrations in urine samples were measured at cycle 1 day 1 predose and cycle 2 day 1 at 0–8 hours postdose. Urine samples were collected from 13 patients (7 patients at 30 mg, 5 at 100 mg, 1 at 250 mg).

### Noncompartmental Analysis

Pharmacokinetic parameters for giredestrant were calculated from the plasma concentration–time data according to standard noncompartmental analysis methods using Phoenix WinNonlin (version 8.3.4; Certara USA, Inc.). Noncompartmental analyses were conducted in pharmacokinetic-evaluable patients, defined as those having at least one nonzero postdose plasma concentration. Patients with dose reductions, sparse pharmacokinetic samples, incomplete pharmacokinetic profiles, or incorrect dosing information during pharmacokinetic sampling were not included in the noncompartmental analysis.

### Bioanalytical Methods

The concentrations of giredestrant in plasma were measured using validated LC/MS-MS assays. Giredestrant and its internal standard [(13C8, 15N) GDC 9545] were extracted from human plasma by supported liquid extraction (SLE). Giredestrant concentrations were calculated using a standard curve with a 1/*x*^2^ linear regression over concentration ranges of 1 to 1,000 ng/mL or 0.1 to 100 ng/mL. The concentrations of giredestrant in urine were measured using a qualified LC/MS-MS assay. Giredestrant and its internal standard were also extracted from human urine by SLE. Giredestrant concentrations were calculated using a standard curve with a 1/*x*^2^ linear regression over a concentration range of 0.25 to 250 ng/mL.

Concentrations of 4β-HC were measured using an LC/MS-MS assay that was developed and validated in human plasma. 4β-HC and its internal standard 4β-HC-d7 were extracted from human plasma by SLE. 4β-HC concentrations were calculated using a standard curve with a 1/*x*^2^ linear regression over a concentration range of 4 to 100 ng/mL.

For plasma, urine, and 4β-HC assays, the mass spectrometer was operated in positive electrospray ionization mode under optimized conditions with multiple reaction monitoring of analytes and internal standards. The precision and accuracy of assays were satisfactory throughout the study.

For metabolite identification, plasma samples were extracted by protein precipitation. Parent and metabolites were analyzed by LC and high-resolution MS. The metabolite structures were proposed on the basis of fragmentation patterns and comparison with those of the parent.

### Food Effect Assessment

In the single-agent dose-escalation stage, giredestrant was taken under fasted conditions (fasted for ≥6 hours overnight) until cycle 4 day 1. To explore whether giredestrant had a potential food effect, patients in the single-agent dose-expansion cohorts were able to take giredestrant with or without food, and were asked to complete a food diary at cycle 2 day 1. Patients who had reported fasting for longer than 6 hours before giredestrant dosing were considered as fasted.

### Ethics

The study was conducted in accordance with the protocol and the consensus ethical principles derived from international guidelines including the Declaration of Helsinki and Council for International Organizations of Medical Sciences International Ethical Guidelines for Health-Related Research Involving Humans, relevant International Conference on Harmonisation Good Clinical Practice guidelines, and applicable laws and regulations. All patients provided written informed consent.

### Data Availability Statement

Phase I studies are not in scope of the Roche global policy on data sharing. Qualified researchers may submit an enquiry through the data request platform, Vivli, https://vivli.org/ourmember/roche/; however, this does not guarantee that the data can be shared. For up-to-date details on Roche's Global Policy on the Sharing of Clinical Information and how to request access to related clinical study documents, see go.roche.com/data_sharing. Anonymized records for individual patients across more than one data source external to Roche cannot, and should not, be linked due to a potential increase in risk of patient reidentification.

## Results

### Study Population

Between November 27, 2017, and January 28, 2021, 175 patients were enrolled in the study: 111 across all single-agent cohorts and 64 in the giredestrant 100 mg combination cohort with palbociclib. Of 175 patients enrolled, 171 were evaluable for pharmacokinetics. In the single-agent dose-escalation phase, there were 29 patients with calculated pharmacokinetic parameters (including 21 with a 7-day single-dose pharmacokinetic lead-in). The data cut-off date was September 17, 2021.

### Pharmacokinetic Characterization

**FIGURE 1:**
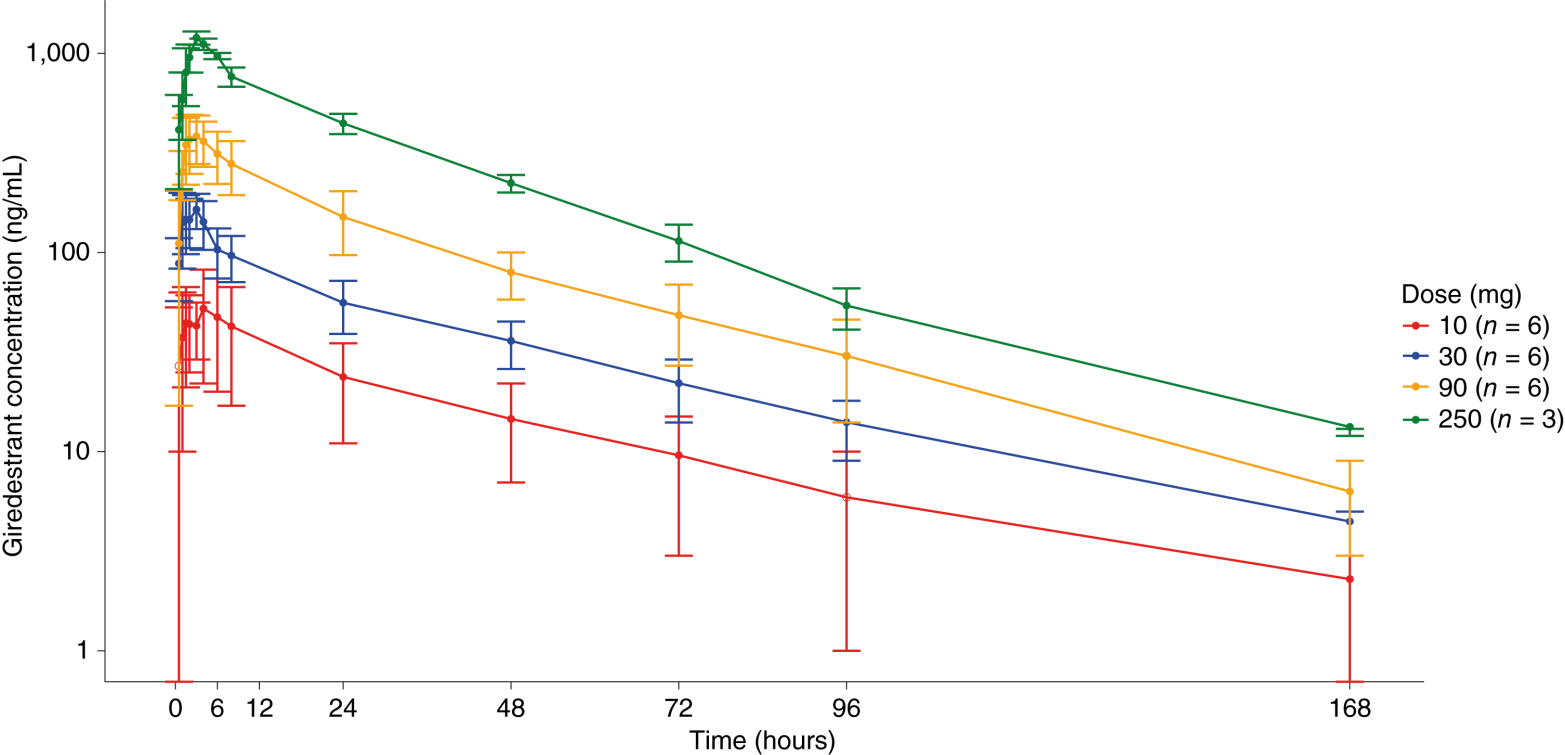
Giredestrant mean (±SD) plasma concentration–time profiles after a single dose.

**FIGURE 2:**
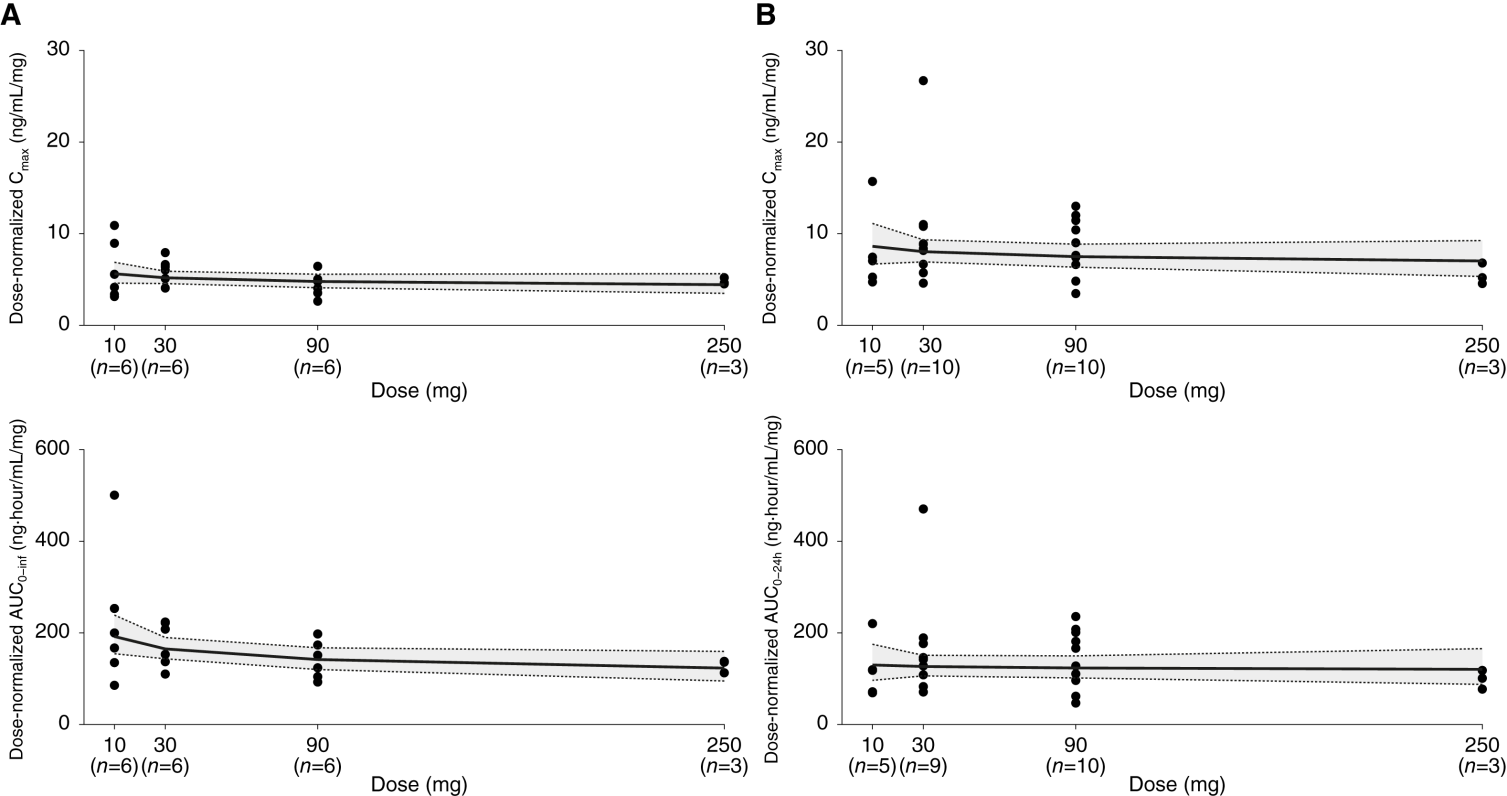
Dose proportionality of giredestrant dose-normalized exposure. **A,** Single-dose C_max_ and AUC_0-inf_. **B,** Steady-state C_max_ and AUC_0–24h_. Solid line represents the linear regression and dotted lines represent the 90% confidence interval from the power model (10). Abbreviations: AUC_0-inf_, area under the plasma concentration–time curve from time 0 extrapolated to infinity. AUC_0–24h_, area under the plasma concentration–time curve from time 0 to 24 hours.

Following oral administration of a single dose under fasted conditions, giredestrant was rapidly absorbed with a median time to maximum concentration (t_max_) of 1.75–3.13 hours across the dose range of 10 to 250 mg (Table 1). The geometric mean half-life after a single dose ranged from 25.8 to 43.0 hours over the same dose range (Table 1; Fig. 1), supporting once-daily dosing. In general, giredestrant showed dose-proportional increases in plasma exposure in the range of 10 to 250 mg, as measured by maximum plasma concentration (C_max_) and area under the plasma concentration–time curve (AUC; ref. 10; Fig. 2). After repeated daily dosing, estimated accumulation ratios were 1.1- to 1.8-fold based on C_max_ and 1.4- to 2.4-fold based on AUC from time 0 to 24 hours (AUC_0–24h_; Table 2).

**TABLE 1:** Giredestrant single-dose pharmacokinetic parameters (escalation cohorts)

Giredestrant dose (No. of patients)	T_max_ (hours)	C_max_ (ng/mL)	AUC_0–24h_ (ng·hour/mL)	AUC_0-inf_ (ng·hour/mL)	T_1/2_ (hours)	V_z_/F (L)	CL/F (L/hour)
10 mg (*n* = 6)	2.49 (0.95–4.17)	53.7 (55.3)	779 (49.1)	1,910 (65.7)	38.9 (32.8)	293 (39.0)	5.23 (65.7)
30 mg (*n* = 6)	1.75 (1.05–3.12)	177 (23.5)	2,100 (26.4)	5,090 (30.1)	43.0 (14.7)	365 (32.4)	5.89 (30.1)
90 mg (*n* = 6)	3.01 (1.52–4.22)	382 (31.8)	5,520 (31.9)	12,200 (30.2)	38.7 (25.3)	411 (37.3)	7.37 (30.2)
250 mg (*n* = 3)	3.13 (3.00–8.03)	1,190 (7.50)	16,100 (8.42)	32,100 (11.3)	25.8 (16.2)	290 (7.71)	7.79 (11.3)

NOTE: Data reported as geometric mean (% CV), except T_max_, which is reported as median and range.

Abbreviations: AUC_0-inf_, area under the plasma concentration–time curve from time 0 extrapolated to infinity; AUC_0–24h_, area under the plasma concentration–time curve from time 0 to 24 hours; CL/F, apparent clearance; T_1/2_, terminal half-life; V_z_/F, apparent volume of distribution during terminal phase.

**TABLE 2:** Giredestrant steady-state pharmacokinetic parameters (escalation cohorts)

Giredestrant dose (No. of patients)	T_max,ss_ (hours)	C_max,ss_ (ng/mL)	AUC_0–24h,ss_ (ng·hour/mL)	C_min,ss_ (cycle 2 day 1 predose) (ng/mL)	AR C_max_	AR AUC_0–24h_
10 mg (*n* = 5)	1.50 (0.967–3.55)	72.8 (49.7)	1,090 (50.0)	30.2 (62.7)	1.56 (37.8)	1.61 (44.0)
30 mg (*n* = 10)	3.00 (1.47–3.83)	266 (50.1)	4,320 (59.4)	130 (65.1)	1.75^a^ (49.2)	2.44^b^ (48.5)
90 mg (*n* = 10)	3.02 (1.50–3.98)	718 (44.3)	11,500 (58.4)	343 (73.4)	1.66^a^ (45.0)	1.82^a^ (55.8)
250 mg (*n* = 3)	3.93 (2.03–4.08)	1,360 (20.6)	24,300 (21.7)	868 (28.2)	1.14 (22.1)	1.37^c^ (10.6)

NOTE: Data reported as geometric mean (% CV), except T_max_, which is reported as median and range.

Abbreviations: AR, accumulation ratio; C_min_, minimum plasma concentration; ss, steady state.

^a^ *n* = 6.

^b^ *n* = 5.

^c^ *n* = 2.

At the clinical dose of 30 mg, the geometric mean [geometric % coefficient of variation (%CV)] of maximum steady-state concentration (C_max,ss_) was 266 ng/mL (50.1%) and the AUC_0–24h_ at steady state (AUC_0–24h,ss_) was 4,320 ng·hour/mL (59.4%; Table 2). The geometric mean (geometric %CV) plasma elimination half-life was 43.0 hours (14.7%).

Metabolite identification from plasma samples suggested that glucuronidation and oxidation are potentially the metabolism pathways for giredestrant (Genentech data on file). No abundant metabolites or long-lived circulating metabolites were identified; specifically, no major oxidation metabolites were seen.

Exploratory assessments of giredestrant concentrations in urine samples over an 8-hour collection period postdose at steady state (cycle 2 day 1) indicated that 0.246% of drug was excreted in urine. Therefore, renal excretion is unlikely to be the major elimination route for giredestrant.

### Intrinsic and Extrinsic Factors

#### DDI Assessment

The mean concentration of 4β-HC showed no apparent increase after multiple doses of giredestrant for up to 84 days (cycle 4 day 1) at both the clinical dose (30 mg) and a supratherapeutic dose (90 mg; Table 3). These results suggest that giredestrant may have low CYP3A induction potential in humans.

**TABLE 3:** Change in 4β-HC concentrations over time, from baseline (cycle 1 day −7) to cycle 4 day 1

		4β-HC (ng/mL)			
Giredestrant dose		Baseline (Cycle 1 day −7)	Cycle 2 day 1	Cycle 3 day 1	Cycle 4 day 1
30 mg	*N*	5	5	4	4
	Mean ± SD	36.9 ± 20.8	38.1 ± 24.0	25.4 ± 10.4	22.0 ± 8.05
90 mg	*N*	2	2	1	1
	Subject 1	72	79	—	—
	Subject 2	42	39	22	25

When giredestrant 100 mg was coadministered with palbociclib 125 mg, giredestrant exposure was generally similar to that observed with single-agent giredestrant (8). Furthermore, the pharmacokinetics of palbociclib when given in combination with giredestrant were consistent with previously reported values with palbociclib alone (8, 11). Coadministration of palbociclib and giredestrant revealed no clinically relevant DDI, indicating that dose adjustment is not necessary when combining these two agents. Full details are reported elsewhere (8).

#### Early Assessment of Food Effect on Giredestrant

**FIGURE 3:**
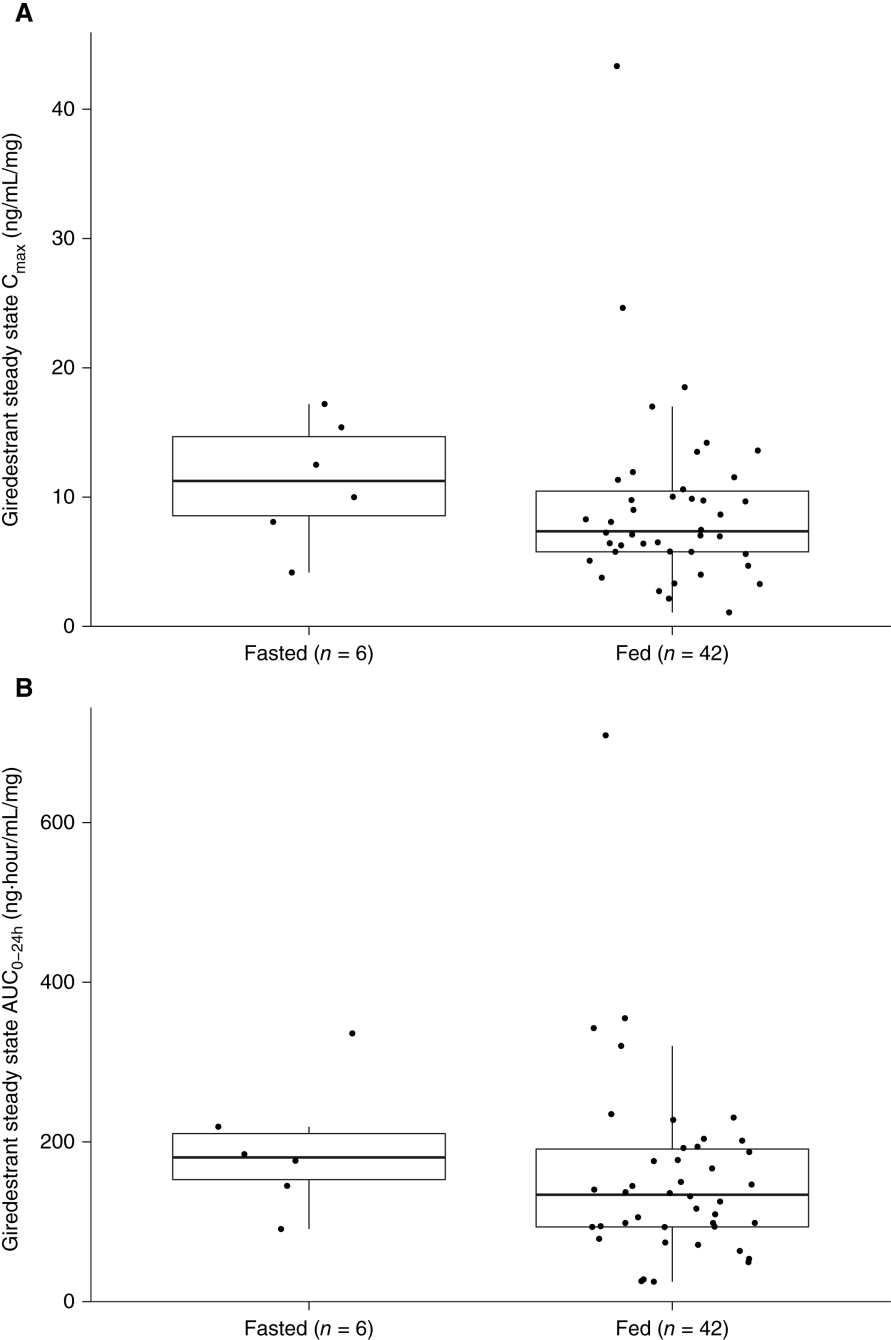
Comparison of dose-normalized giredestrant steady-state exposure between fasted and fed patients in single-agent dose-escalation and -expansion cohorts. **A,** C_max_. **B,** AUC_0–24h_. Pharmacokinetic parameters were dose normalized by the dose received at the time of pharmacokinetic sampling—AUC_0–24h_ or C_max_/Dose. The solid line in the center represents the median, the solid line at the bottom is the 25th percentile (Q1), the solid line at the top is the 75th percentile (Q3), the boxes indicate the interquartile range (IQR; Q1–Q3), and the whiskers represent 1.5*IQR.

The exploratory food effect assessment conducted in the single-agent dose-escalation and dose-expansion cohorts showed no remarkable differences in steady-state exposure between fasted and fed patients (Fig. 3).

#### Race

**FIGURE 4:**
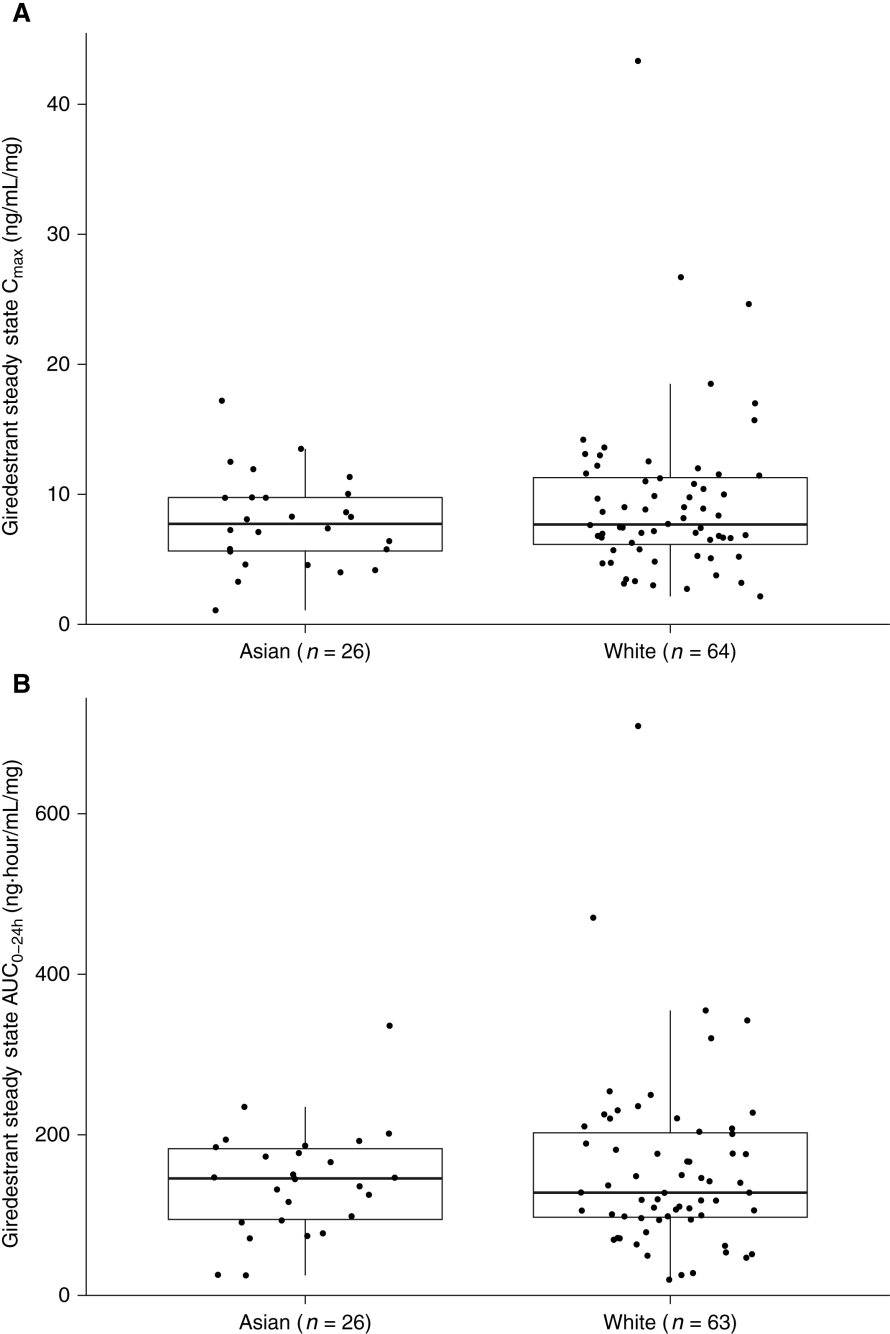
Comparison of dose-normalized giredestrant steady-state exposure between Asian and White patients in escalation and expansion cohorts. **A,** C_max_. **B,** AUC_0–24h_. Pharmacokinetic parameters were dose normalized by the dose received at the time of pharmacokinetic sampling—AUC_0–24h_ or C_max_/Dose. The solid line in the center represents the median, the solid line at the bottom is the 25th percentile (Q1), the solid line at the top is the 75th percentile (Q3), the boxes indicate the IQR (Q1–Q3), and the whiskers represent 1.5*IQR.

In the preliminary assessment of the impact of race on giredestrant exposure conducted in the dose-escalation and dose-expansion stage, giredestrant exposures were generally consistent between Asian and White patients (Fig. 4).

## Discussion

This phase Ia/Ib dose-escalation/-expansion study illustrates opportunities in oncology drug development from a clinical pharmacology perspective, where integration of clinical pharmacology considerations into early-phase clinical trials can accommodate an accelerated oncology development timeline by providing insights for patient eligibility, concomitant medications, and combination regimens for late-stage studies. The enhanced understanding of the pharmacokinetic profile, dose linearity, metabolic pathway, food effect, DDI potential, impact of race, and appropriate dosing when giredestrant is administered with palbociclib informed the design of pivotal trials.

The proposed starting dose for this study was 10 mg once daily, which was projected to be efficacious based on results from nonclinical xenograft models and is approximately 10-fold lower than the calculated maximum recommended starting dose based on the severely toxic dose in 10% of animals from a rat toxicity study (Genentech data on file). In this study, we characterized pharmacokinetics over a wide dose range (10–250 mg) with sufficient exposure separation across different dose levels. Furthermore, the interindividual pharmacokinetic variability observed was in line with that of small molecule drugs in oncology patients (12). Giredestrant demonstrated rapid oral absorption that was generally dose-proportional over the dose range evaluated. Furthermore, giredestrant achieved higher plasma concentration than fulvestrant, an approved SERD given as an intramuscular injection due to its low bioavailability. To optimize dose selection, three dose levels (30, 100, and 250 mg) were evaluated in the expansion cohort to characterize the safety and clinical activity further and the totality of data was evaluated to select the recommended phase II dose.

[^18^F]-fluoroestradiol PET indicated a high degree of target engagement at all dose levels, including 30 mg (8). In addition, analysis of circulating tumor DNA in the *ESR1*-mutant population showed a consistent reduction in *ESR1* variant allele frequency at the 30 mg dose, which was not enhanced at higher doses (8). Doses above 30 mg provided no additional benefit as measured by clinical benefit rate (8). Furthermore, bradycardia was a dose-dependent adverse reaction of giredestrant and was more frequent at doses greater than 30 mg (8). The clinical pharmacology attributes, clinical activity, and safety, together with nonclinical data, supported selection of the 30 mg dose for further development of giredestrant in patients with metastatic and early ER^+^ breast cancer (7, 8, 13). Full details of the dose selection rationale will be reported separately.

The study design also included early assessment of metabolism and excretion through metabolite identification and measurement of giredestrant concentration in urine. The exploratory metabolite identification indicated that an apparently prominent metabolite in humans was absent, which reduced the risk of metabolite-related safety concerns and DDI. The observed minimal renal elimination of giredestrant suggests that renal function is unlikely to have a clinically relevant impact on giredestrant exposure and that the risk for renal transporter-related DDIs with giredestrant is low.

*In vitro* data suggested that giredestrant may induce CYP3A mRNA (Genentech data on file). In the current study, 4β-HC was measured at both the clinical dose (30 mg) and a supratherapeutic dose (90 mg) to enable further evaluation of CYP3A induction potential. 4β-HC has been suggested as an endogenous biomarker for assessing *in vivo* CYP3A activity due to the low variability in its plasma concentration over time. Furthermore, concentrations of 4β-HC have increased after treatment with several strong CYP3A inducers, as well as moderate (efavirenz) and weak (ursodeoxycholic acid) CYP3A inducers (9). The exploratory assessment elucidated that there was no apparent increase in 4β-HC levels from baseline over a prolonged period. Furthermore, baseline 4β-HC was within the range observed in previous studies (9). The prolonged sample collection period was informative because 4β-HC response may be delayed after CYP3A induction due to the long half-life of 4β-HC. Although the sample size of the analysis was limited, the negative finding provides additional evidence that giredestrant may have low CYP3A induction risk.

We found no clinically relevant DDI between giredestrant and palbociclib, a CYP3A substrate. The low DDI potential observed in this phase I study supported phase III evaluation of giredestrant combined with palbociclib as first-line therapy for metastatic breast cancer in the persevERA trial (NCT04546009). In contrast, another SERD, amcenestrant, induced CYP3A at therapeutic dose levels, with increasing induction observed with increasing dose (14). The DDI finding resulted in evaluation of reduced-dose amcenestrant with palbociclib in the (recently discontinued) AMEERA-5 trial (NCT04478266). These findings highlight differences in clinical pharmacology attributes between oral SERD candidates.

The exploratory food effect assessment in this phase I study showed a lack of food effect on giredestrant exposure and guided the dosing recommendation regarding meal intake in late-stage studies. The exploratory food effect assessment was based on patient-reported food diaries, which provided a convenient method for collecting patient meal information; however, the precision of food diaries depends on the accuracy of patient reporting and may not capture exact details of the timing or amount of each patient's caloric and fat consumption. The lack of food effect was subsequently confirmed in a dedicated food effect study in healthy subjects (NCT04274075). From a practical perspective, the absence of a food effect on pharmacokinetics offers greater flexibility and improves patient convenience. Among the other SERDs in clinical development a food effect was observed with elacestrant and rintodestrant. Specifically, it appears food was used to improve rintodestrant bioavailability (7) and the package insert for elacestrant stipulates to take elacestrant with food to improve gastrointestinal tolerability (15).

A unique aspect of this phase I study was the preliminary assessment of the impact of race on giredestrant exposure. Early-phase clinical trials often enroll a less diverse population (16). There are examples where racial differences in efficacy and tolerability have exposed patients to unacceptable toxicity or reduced efficacy (17–19). The current phase I study was performed globally, and included a substantial number of Asian and White patients. This approach allowed us to compare exposure in Asian versus White patients, thus reducing the need for a subsequent clinical bridging study between these two populations. Although the current study provided limited data from Black or Hispanic patients, we plan to investigate these populations further through population pharmacokinetic analysis in larger and more diverse studies, such as the phase III lidERA trial (NCT04961996), which has a broader geographical footprint including sites in Africa and Central and South America.

Insights generated in the current study informed phase II [aceIERA (NCT04576455; ref. 20) and coopERA (NCT04436744; ref. 21)] and phase III (persevERA and lidERA) trial designs. By carefully integrating clinical pharmacology characterization into this first-in-human study, we were able to support accelerated clinical development, with the hope of bringing a new agent to patients more rapidly.

## Supplementary Material

Table S1 Representativeness of study population

Figure S1 Study design. LHRH, luteinizing hormone-releasing hormone.
