# Supplementary material for: Optimizing Early-stage Clinical Pharmacology Evaluation to Accelerate Clinical Development of Giredestrant in Advanced Breast Cancer
Source: Cancer Res Commun. 2023 Dec 15;3(12):2551–9. doi: 10.1158/2767-9764.CRC-23-0324 (PMC10722959; doi:10.1158/2767-9764.CRC-23-0324)
Supplement: Table S1 — Representativeness of study population [file crc-23-0324-s01.pdf]

Supplementary Data

Table S1. Representativeness of study participants.

Cancer type(s)/subtype(s)/stage(s)/condition	Estrogen receptor-positive, HER2-negative, locally advanced/metastatic breast cancer
Considerations related to:	
Sex	Breast cancer is mostly diagnosed in females (~99% of cases in the USA) and is rare in males (1). The estimated global incidence in 2020 was 2,261,419 new cases; accounting for 11.7% of all cancers (2, 3).
Age	The median age at diagnosis for breast cancer is 62 years in the USA; White women tend to be diagnosed at a slightly older age (64 years) than Hispanic, Asian/Pacific Islander, Black, and American Indian/Alaska Native women (57, 58, 60, 61 years, respectively) (1).
Race/ethnicity	White women are slightly more likely to develop breast cancer compared with Black, Hispanic, or Asian women. However, Black women have a 40% higher breast cancer mortality rate than White women in the USA (1).
Geography	Asia has the highest breast cancer incidence (1,026,171 versus 531,086 in

	Europe; 281,591 in North America; 210,100 in Latin America/the Caribbean; 186,598 in Africa; and 25,873 in Oceana) (3).<br>Similarly, Asia has the highest number of deaths from breast cancer (346,009 versus 141,765 in Europe; 85,787 in Africa; 57,984 in Latin America/the Caribbean; 48,407 in North America; and 5,044 in Oceania) (3).
Other considerations	Around 70%–80% of breast cancer cases are estrogen receptor-positive (4, 5), and around 85%–80% are HER2-negative (6).
Overall representativeness of this study	We did not enroll any male patients, as breast cancer is rare in men.<br>The median age of patients in our study was similar to the reported global medians (57–60 years across cohorts).<br>We enrolled no Black patients; our study population was limited to American Indian/Alaska Native, Asian, White, and Unknown races, and the majority of patients were White.<br>Patients were enrolled from Australia, Europe (Spain, United Kingdom), Asia

	(Republic of Korea) and North America (United States).

Supplemental references

1. Giaquinto AN, Sung H, Miller KD, Kramer JL, Newman LA, Minihan A, *et al*. Breast cancer statistics, 2022. *CA Cancer J Clin* **2022**;72:524-41.
2. Globocan. All cancers. [cited 2023 May]. Available from: https://gco.iarc.fr/today/data/factsheets/cancers/39-All-cancers-fact-sheet.pdf.
3. Globocan. Breast. [cited 2023 May]. Available from: https://gco.iarc.fr/today/data/factsheets/cancers/20-Breast-fact-sheet.pdf.
4. Freelander A, Brown LJ, Parker A, Segara D, Portman N, Lau B, *et al*. Molecular biomarkers for contemporary therapies in hormone receptor-positive breast cancer. *Genes (Basel)* **2021**;12:285.
5. Lumachi F, Santeufemia DA, Basso SM. Current medical treatment of estrogen receptor-positive breast cancer. *World J Biol Chem* **2015**;6:231-9.
6. Wolff AC, Hammond EH, Hicks DG, Dowsett M, McShane LM, Allison KH, *et al* Recommendations for human epidermal growth factor receptor 2 testing in breast cancer: American Society of Clinical Oncology/College of American Pathologists clinical practice guideline update. *J Clin Oncol* **2013**;31:3997-4013.
