# Supplementary material for: Optimizing Early-stage Clinical Pharmacology Evaluation to Accelerate Clinical Development of Giredestrant in Advanced Breast Cancer
Source: Cancer Res Commun. 2023 Dec 15;3(12):2551–9. doi: 10.1158/2767-9764.CRC-23-0324 (PMC10722959; doi:10.1158/2767-9764.CRC-23-0324)
Supplement: Figure S1 — Study design. LHRH, luteinizing hormone-releasing hormone. [file crc-23-0324-s02.pdf]

Supplementary Figure S1

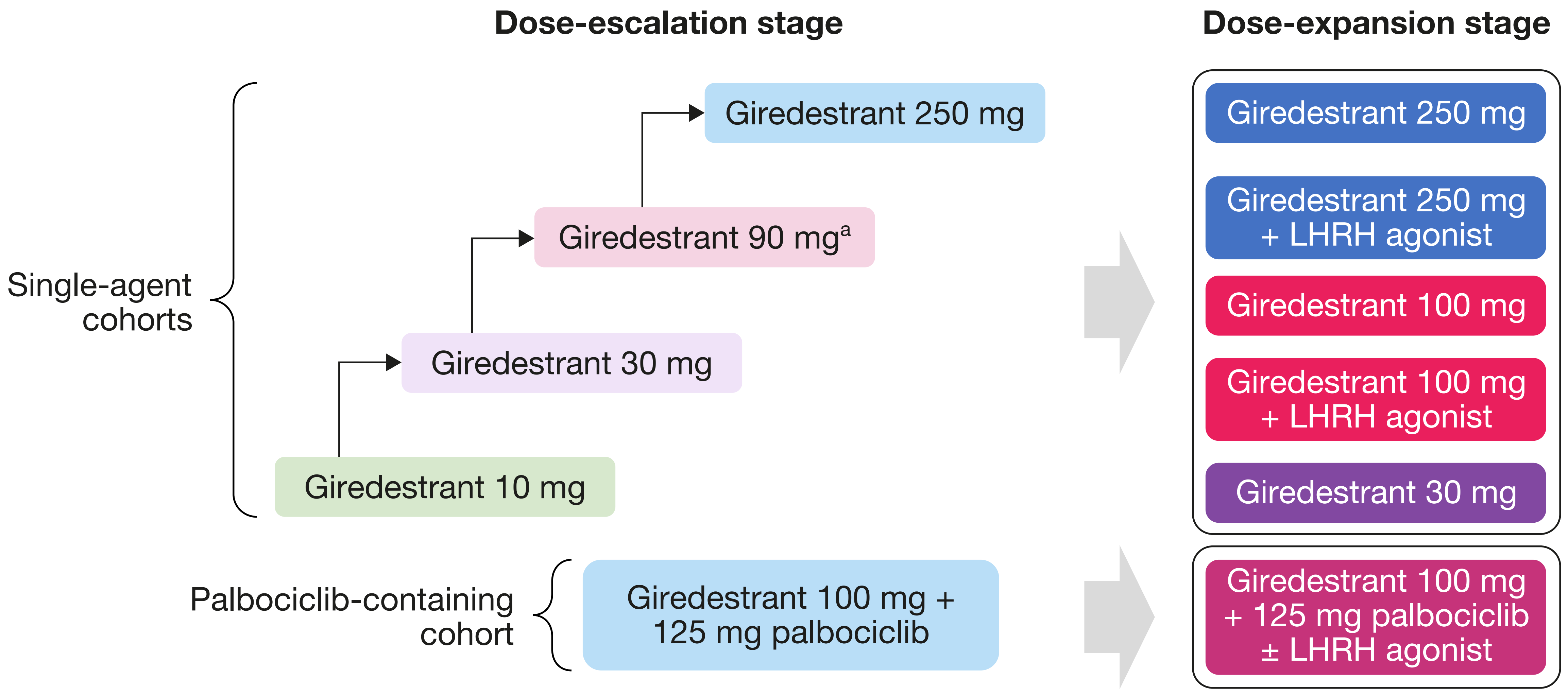

[a]The 90 mg dose originally assigned in the escalation stage was adjusted to 100 mg for ease of dosing and considered equivalent to the 90 mg dose.
